# Fungal Community Structure and Diversity in Four Habitat Substrates at Pied Avocet (*Recurvirostra avosetta*) Breeding Sites of the Yellow River Delta Coastal Wetlands

**DOI:** 10.3390/biology15131015

**Published:** 2026-06-26

**Authors:** Xinping Yu, Qinghua Cui, Bo Zhou, Jingyi Yu, Shichang Liu, Yaojia Cao, Shuai Shang, Jun Wang, Yunpeng Liu

**Affiliations:** 1 College of Biological and Pharmaceutical Engineering, Shandong University of Aeronautics, Binzhou 256600, China; 15063135251@163.com (X.Y.); 15085829033@163.com (B.Z.); yujingyi010815@126.com (J.Y.); m18669498261@163.com (S.L.); cyjj345678@163.com (Y.C.); shangshuai8983@126.com (S.S.); ivywangjun@163.com (J.W.); 2 Institute of Environmental Protection Science and Technology of Binzhou, Binzhou 256600, China; qhcui1981@163.com; 3 Shandong Key Laboratory of Eco-Environmental Science for the Yellow River Delta, Binzhou 256600, China

**Keywords:** shell dykes, *Recurvirostra avosetta*, fungi, Yellow River Delta

## Abstract

To understand how habitat heterogeneity drives fungal community assembly in different habitats of the pied avocet (*Recurvirostra avosetta*), we analyzed four habitat types (water bodies, aquatic plants, soil, and nest sediments) using high-throughput sequencing. A total of 9980 ASVs (Amplicon Sequence Variants) were detected, with only 68 shared across all habitats, indicating strong community differentiation. Ascomycota and Basidiomycota dominated (50–60% relative abundance), reflecting fungal adaptability to wetlands. Water bodies showed significantly higher alpha diversity than aquatic plants and nest sediments. Beta diversity and principal coordinates analysis (PCoA) revealed closer similarity in fungal composition between water and aquatic plant communities, whereas soil and nest sediments formed distinct clusters. PERMANOVA based on binary Jaccard distances further confirmed that habitat type explained 10.9% of the variation in fungal community structure (R^2^ = 0.109, *p* = 0.001). LEfSe (LDA Effect Size) identified habitat-specific indicator taxa, supporting niche filtering and competitive exclusion as selection mechanisms. The co-occurrence network was dominated by positive correlations, suggesting metabolic complementarity that maintains ecosystem stability. Unclassified fungi accounted for 18–22% of communities, representing untapped fungal resources. These findings support that habitat heterogeneity governs multi-media fungal assembly, revealing how microhabitat conditions regulate fungal composition, diversity, and interactions. This study provides a theoretical basis for biodiversity conservation and ecological restoration in avocet habitats.

## Simple Summary

The pied avocet is a wetland bird whose breeding success is closely tied to the condition of its surrounding environment. In this study, we investigated the fungal communities present in four distinct components of the avocet’s breeding habitat in the Yellow River Delta: open water, aquatic plants, soil, and nesting sediments. Using DNA-based identification, we found that each component harbored a largely distinct set of fungi, with only a small fraction shared across all habitats. This pattern indicates that fungal communities are highly sensitive to local environmental conditions, and that even subtle differences in habitat type can lead to marked shifts in species composition. We also observed that the fungi within each habitat formed complex networks dominated by cooperative interactions, which likely contribute to the overall stability and functioning of the wetland ecosystem. A large proportion of detected fungi could not be classified, indicating significant undiscovered fungal diversity in these wetlands. Our results highlight the importance of maintaining a mosaic of habitat types, not just open water, to support the full range of fungal communities. Preserving this diversity sustains ecological processes that support avocet breeding grounds’ health, offering practical guidance for wetland conservation and restoration.

## 1. Introduction

The Binzhou Shell Dykes are located in the sedimentary belt of the Yellow River Delta, where a broad coastal wetland and a tidal flat landform resembling a shell beach ridge system have developed over thousands of years [1]. As a distinctive geomorphological feature of the Yellow River Delta, the shell dyke ecosystem serves as a key research area for understanding coastal wetland formation, biodiversity maintenance, and fungal ecology. When the river diverts or sediment transport declines markedly, intensified wave–tide interactions promote the shoreward migration of shell debris, whose gradual accumulation forms shell embankments [2]. This region serves as a vital stopover along the inland Northeast Asian and Western Pacific Rim bird migration routes, providing critical habitats for wintering, roosting, and breeding [3].

The Pied Avocet (*Recurvirostra avosetta*), a member of the family Recurvirostridae, is a key wetland species that performs irreplaceable ecological functions and acts as a sensitive indicator of ecosystem health [4]. By filter-feeding on small crustaceans, it regulates prey populations and sustains wetland ecological balance. Its foraging activity also stirs sediments, facilitates material exchange, elevates dissolved oxygen, and accelerates organic decomposition. Evidently, the Pied Avocet exerts positive effects on supporting diverse species and enriching wetland biodiversity [5,6]. Its distinctive morphology, behavior, and migratory patterns offer valuable models for evolutionary biology and wetland ecology, informing conservation and management strategies [7]. As the core interface of environmental interaction, the intestinal microbiota of the Pied Avocet is closely tied to material cycling, energy flow, and ecological equilibrium in bird habitats. Unraveling how this microbiota responds to ecosystem changes can enrich coevolutionary theory, shed light on biogeochemical processes, and underpin comprehensive wetland protection systems [8,9].

Currently, research on microorganisms in the habitats of waders, including the Pied Avocet, remains very limited. Existing studies have mostly focused on fungal community analyses in single media such as soil or water, lacking systematic investigations into the correlations and synergistic relationships among fungal communities across multiple media, including nest sediments, water, soil, and aquatic plants [8,10]. Although feces are a direct product of avian activity and an important carrier reflecting interactions with the habitat [11], fresh feces in field conditions are highly susceptible to physical weathering and fungal degradation, leading to rapid changes in their fungal composition and physicochemical properties, which compromises the stability and accuracy of analytical results [12]. Therefore, relying directly on fecal samples makes it difficult to accurately and stably reflect the impact of avian activity on the habitat fungal system, and there is an urgent need to explore more reliable research approaches [12,13]. To circumvent the instability and sampling challenges associated with fresh feces, this study proposes a method for extracting fungal information from core multi-media components of the wetland ecosystem (nest sediments, water, soil, and aquatic plants) and conducting cross-validation among these media [8]. This approach aims to more objectively reveal the complex interactions between Pied Avocet activity and the habitat fungal system. This strategy not only fills the gap left by traditional single-media studies but also avoids the limitations of fecal analysis, significantly improving the feasibility of the research and the reliability of the results [14]. Given the difficulty of collecting fresh avocet feces, this study employs the combined fungal profiles of nest sediments, water, soil, and aquatic plants to indirectly infer the influence of Pied Avocet activity on the habitat system [8,15], thereby providing a new research perspective and methodological support for exploring the interactions between waders and fungal ecosystems.

This study selected the Binzhou Shell Dike Island as the research area, which represents a typical natural shell ridge wetland ecosystem within the Yellow River Delta. We systematically collected four types of media samples (nest sediments, water, soil, and aquatic plants) and employed high-throughput sequencing to characterize fungal community structure, aiming to compare fungal community differences across media types, identify shared and unique taxa, and elucidate the potential influence of Pied Avocet activity on habitat fungal communities. By integrating multiple analytical approaches, this study clarified the potential environmental sources of fungal communities in nest sediments and assessed the ecological effects of fungal dispersal between nesting microhabitats and surrounding environments. The sampling design and sample labeling system in this study are fully consistent with those used in the bacterial investigation reported by Yu et al. (2026) [8], ensuring data comparability for subsequent cross-kingdom comparisons; however, the scientific questions, core hypotheses, sequencing targets (ITS vs. 16S rRNA), and analytical framework of this fungal study are entirely independent from the bacterial study. Focusing on the Pied Avocet, we established a multi-substrate comparative framework that overcomes the limitations of single-substrate sampling designs and specifically targets the functionally distinctive fungal communities that are often overlooked in conventional microbial surveys. At the theoretical level, this study advances fundamental understanding of fungal ecology from three key dimensions. First, in contrast to bacterial communities, which are primarily driven by local environmental filtering, fungal community assembly in Pied Avocet habitats exhibits complementary mechanisms, shaped by the synergistic effects of airborne spore dispersal, substrate-specific decomposition pathways, and host-mediated selective pressures. Second, pairwise comparisons across the four media types (water, aquatic vegetation, soil, and nest sediments) demonstrated that media type exerts a much stronger influence on fungal β-diversity than spatial distance—a pattern distinctly different from bacteria, whose biogeographic distribution is primarily constrained by dispersal limitation. Third, cross-media co-occurrence network analyses revealed habitat-specific fungal indicator taxa, including saprotrophic Ascomycota and yeast-producing Basidiomycota, which can serve as sensitive bioindicators for tracking habitat humidity dynamics and organic matter cycling, providing functional insights that cannot be obtained from bacterial analyses alone. From an applied perspective, these fungi-centric findings provide additional microbiological evidence for the conservation management of the Binzhou Shell Dike Island Wetland National Nature Reserve; notably, fungal communities may respond to environmental stressors earlier than bacterial communities, making them potentially valuable early-warning bioindicators for monitoring habitat degradation.

## 2. Materials and Methods

### 2.1. Sample Collection and Study Area

**Figure 1:**
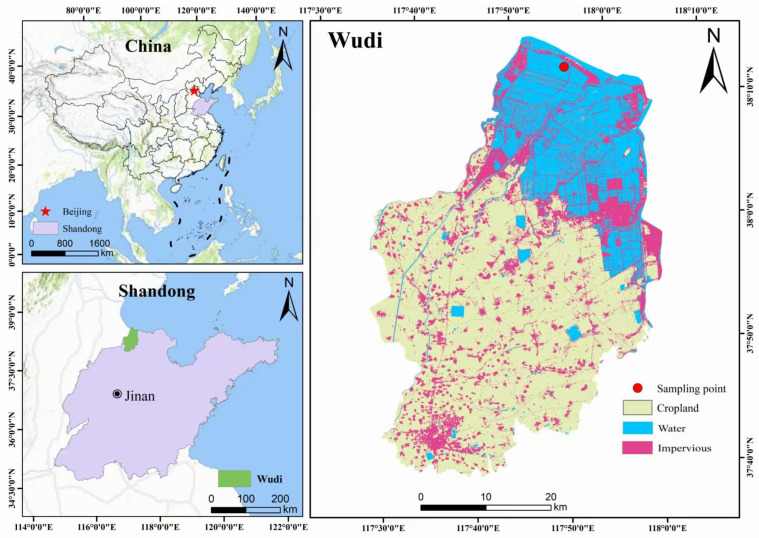
Geographical map of the sampling area (Reproduced from Yu et al., 2026, *Front. Ecol. Evol.* 14, 1841914) [8]. (The black dashed line represents China’s nine-dashed line in the South China Sea, marking the traditional maritime boundary of China in the South China Sea).

The study area encompasses the Binzhou Shell Dyke Island and Wetland National Nature Reserve in the Yellow River Delta, located in Wudi County, Binzhou City, Shandong Province, on the southwestern coast of Bohai Bay (38°02′50.51″–38°21′06.06″ N, 117°46′58.00″–118°05′42.95″ E). The specific sampling site was situated at 117°55′17.97″ E, 38°13′28.16″ N, consistent with the companion bacterial study (Yu et al., 2026) [8] (Figure 1). This region experiences a warm-temperate, semi-humid continental monsoon climate with pronounced marine and monsoonal influences [16]. The climate features four distinct seasons: hot, rainy summers with concentrated precipitation; cold, dry winters; and short spring and autumn periods, with spring being particularly windy and arid [17,18]. The landscape is dominated by shell-sand beaches, which provide important breeding and foraging habitats for the Pied Avocet [3,19]. Sampling was conducted in early September 2025, approximately 1–2 months after chick fledging. While fresh feces were unavailable, nest sediments collected at this time represent organic material that accumulated during the breeding period, including feathers, food remains, and excreta from both adult and chick activities [7]. A representative core sampling area (approximately 2 km × 2 km) was established within the main activity zone of the Pied Avocets, located adjacent to the eastern side of an earth embankment road (approximately 117°55′ E, 38°08′ N). Ten nest sites were selected across the core breeding area. These nests were arranged in a clustered distribution, forming several breeding patches. Within the same patch, adjacent nests were separated by ≥5 m to capture fine-scale spatial heterogeneity; between different patches, the inter-patch distance was approximately 50 m to ensure spatial independence. Around each nest, a sampling unit comprised three spatially distinct sub-units: (1) directly beneath the nest (nest sediment), (2) within 0–1 m from the nest edge (nest-associated microhabitat), and (3) the surrounding foraging area (10–20 m from the nest). Each sampling unit thus represented the immediate surrounding environment of an individual nest, as well as broader habitat types associated with the avocet breeding area, including the shell-sand foraging flats. Within each sampling unit, all four types of environmental media were systematically collected: nest sediments (surface sediments collected at 0–5 cm depth directly beneath the nest structure), water samples (surface water collected from temporary pools within the foraging area), soil samples (mineral soil layers collected at 0–10 cm depth beneath the surface in foraging areas), and aquatic plant samples. All environmental media were sampled in parallel across the ten sampling units. Sterile sampling tools and containers, including water samplers, shovels, scissors, sterile sampling bags, sterile sampling bottles, and sterile gloves, were used throughout the procedure. All relevant information was recorded on standardized sampling record sheets. Nest sediment samples were labeled FZC1 to FZC10, water samples FZL1 to FZL10, soil samples FZT1 to FZT10, and aquatic plant samples FZS1 to FZS10. All samples were immediately transported to the laboratory and stored at 4 °C pending further analysis.

### 2.2. DNA Extraction, PCR Amplification, Sequencing, and Bioinformatics Analysis

Total genomic DNA was extracted from all samples at BioMed Biotechnology Co., Ltd. (Qingdao, Shandong, China) using the Tiangen S96 Magnetic Soil and Fecal DNA Kit (Tiangen Biotech Co., Ltd., Beijing, China) following the manufacturer’s instructions. The fungal internal transcribed spacer (ITS) region was amplified with fungal-specific primers, and the resulting amplicons were purified, quantified, pooled equimolarly, and sequenced on the Illumina NovaSeq 6000 platform (Illumina Inc., San Diego, CA, USA) in paired-end mode. Raw reads were processed with Cutadapt (Cutadapt 1.9.1, TU Dortmund University, Dortmund, Germany) to remove primer sequences, followed by quality filtering with Trimmomatic (Trimmomatic v0.33, Max Planck Institute of Molecular Plant Physiology, Potsdam-Golm, Germany) (sliding window 4 bp, average quality ≥ Q20, minimum read length 36 bp). Filtered sequences were denoised using the DADA2 algorithm (v1.20, Department of Statistics, Stanford University, Stanford, CA, USA) in QIIME 2 (QIIME2 2020.6, QIIME 2 Development Team, Boulder, CO, USA) [17,20]: forward and reverse reads were truncated at quality-score drops below Q20, reads with expected errors > 2.0 were discarded, paired ends were merged with a minimum overlap of 12 bp and zero mismatches, and chimeras were removed by the consensus method [19,20], yielding high-quality amplicon sequence variants (ASVs). Taxonomy was assigned against the UNITE database (v9.0) using the Naïve Bayes classifier with a confidence threshold of 0.7, and ASVs unassigned by UNITE were cross-verified by BLAST (v2.14.0, National Center for Biotechnology Information, Bethesda, MD, USA) against NCBI GenBank(National Center for Biotechnology Information, Bethesda, MD, USA); non-fungal sequences were manually removed. A Venn diagram was constructed using the R package VennDiagram (v1.7.3) to display shared and unique ASVs across media [21]. Community composition was visualized at phylum, class, order, family, and genus levels with bar plots (ggplot2 v3.5.0) and heatmaps (pheatmap v1.0.12) [22,23]. Alpha diversity was assessed using the Chao1 richness estimator and Simpson diversity index (vegan v2.6-4) after rarefaction to the minimum sequencing depth, and rarefaction curves and rank-abundance curves were generated with vegan and ggplot2 [24]. Beta diversity was analyzed based on binary Jaccard distance matrices, chosen because presence–absence data are less affected by dormant propagules, PCR bias, and rare ASVs, and to maintain analytical consistency with the companion bacterial study [8]. Ordination was visualized via principal coordinate analysis, non-metric multidimensional scaling, and clustering heatmaps (phyloseq v1.46.0, vegan v2.6-4) [25,26]. Permutational multivariate analysis of variance (PERMANOVA, adonis2, 999 permutations) based on binary Jaccard distances confirmed significant differences among habitat types (R^2^ = 0.109, *p* = 0.001) [27,28]. Differentially abundant ASVs and taxa were screened across taxonomic levels to identify biomarkers [14]. Co-occurrence networks were constructed using ASVs with > 0.1% relative abundance detected in at least three samples; pairwise Spearman’s rank correlations and Benjamini–Hochberg corrections were computed with the R package Hmisc, and significant correlations (|ρ| > 0.6, adjusted *p* < 0.05) were retained for network visualization with Gephi (v0.10.1) and igraph (v1.4.x). Node size was scaled to mean relative abundance, positive edges were shown as solid red lines and negative edges as dashed green lines, and hub taxa were identified by node degree and betweenness centrality.

## 3. Results

### 3.1. Multi-Media Fungal ASV Abundance Analysis

**Figure 2:**
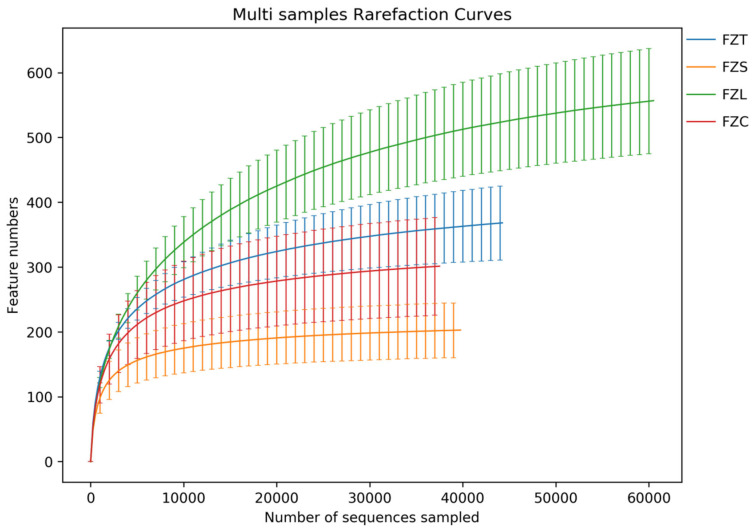
Multi-sample Rarefaction Curves. FZC, FZS, FZL and FZT represent nest sediment, aquatic plant, water and soil samples, respectively.

Rarefaction curve analysis [29] revealed that the curves for all sample groups plateaued with increasing sequencing depth (Figure 2). After approximately 30,000 sequences, the curves leveled off, with clearly reduced increments. The plateau heights of the four groups differed and ranked in descending order as follows: FZL > FZT > FZC > FZS. Specifically, the FZL group exhibited the highest plateau level (approximately 570 features), while the FZS group showed the lowest (approximately 320 features).

**Figure 3:**
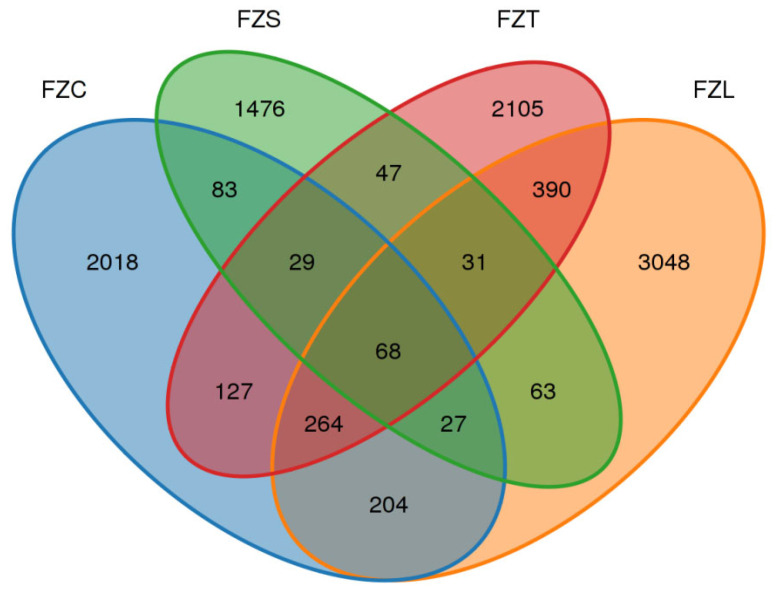
Venn diagram illustrating fungal ASV composition of four habitats: FZC (nest sediments), FZS (aquatic plants), FZL (water), and FZT (soil). Values denote counts of unique or shared ASVs, revealing clear divergence in fungal communities across habitats.

Venn diagram analysis based on the feature sequences of the four sample groups FZC, FZS, FZT, and FZL revealed that each group harbored unique features (Figure 3). Among them, the FZL group contained the highest number of unique features (3048), followed by FZT (2105), FZC (2018), and FZS (1476). The number of pairwise shared features varied considerably among groups, with the highest sharing observed between FZT and FZL (390 features) and the lowest between FZC and FZS (83 features). The remaining pairwise intersections FZC–FZT, FZC–FZL, FZS–FZT, and FZS–FZL—comprised 127, 204, 47, and 63 features, respectively. The number of features shared exclusively by three groups was generally low, with the triple intersections FZC–FZS–FZT, FZC–FZS–FZL, FZC–FZT–FZL, and FZS–FZT–FZL containing 29, 27, 264, and 31 features, respectively. A total of 68 core features were shared across all four groups, representing the stable common components present in all treatments.

### 3.2. Alpha Diversity Analysis of Multi-Media Fungal Community

**Figure 4:**
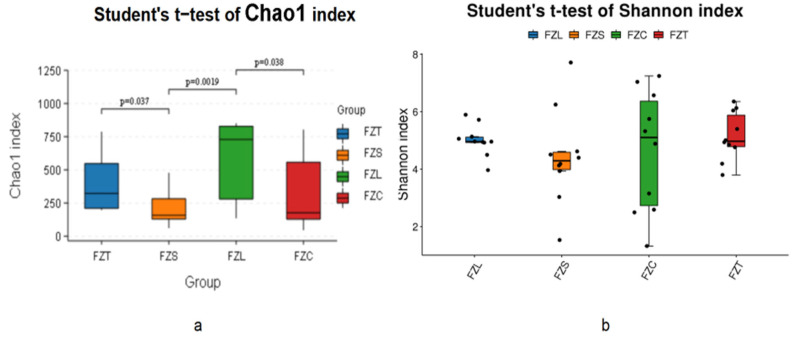
Alpha diversity of fungal communities in different habitat types. Box plots showing (**a**) Chao1 index (reflecting species richness) and (**b**) Shannon index (comprehensively reflecting species diversity and evenness). The central line represents the median; box limits represent the interquartile range (IQR); the error line represents the maximum and minimum values. Asterisks indicate significant differences between groups.

The Chao1 index (Figure 4a) revealed that water samples (FZL) exhibited the highest species richness, with a median of approximately 720. This value was significantly higher than those of aquatic plants (FZS, Student’s *t*-test, *p* = 0.0019), nest sediments (FZC, *p* = 0.038), and soil (FZT, *p* = 0.037). Species richness among aquatic plants, nest sediments, and soil did not differ significantly from one another.

The Shannon diversity index (Figure 4b) also showed differences among the four media. Nest sediment (FZC) and soil (FZT) samples had median Shannon values of approximately 5.2 and 5.1, respectively, which were significantly higher than those of water (FZL) and aquatic plants (FZS). The Shannon index of water samples exhibited a narrower distribution, whereas aquatic plant samples showed wider variability.

### 3.3. Analysis of Multi-Media Fungal Community Structure

**Figure 5:**
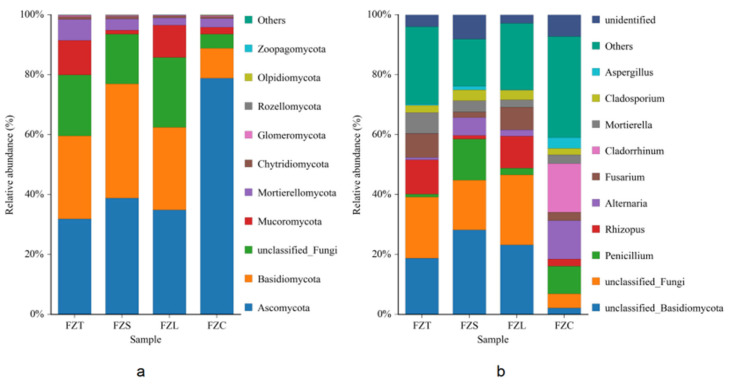
Relative abundance histograms of fungal communities at the phylum level (**a**) and the genus level (**b**) in different media of the Pied Avocet habitat are presented. (**a**) is a histogram of relative abundance at the phylum level, depicting the relative abundance distribution of fungal communities in water samples (FZL), water plants (FZS), soil samples (FZT), and nest sediments (FZC) at the phylum level. Different colors represent different phyla. (**b**) is the relative abundance histogram at the genus level, showing the relative abundance distribution of fungi in four media, and different colors represent different genera. The data in the figure are represented by the average value, with only the top ten species in terms of abundance level shown, and other species are merged into “Others”. In the figure, “unclassified” represents the species that have not been taxonomically annotated, and the specific species information can be found in the species abundance table at the corresponding classification level.

Fungal communities in the four habitat media of the Pied Avocet exhibited compositional differences at both the phylum and genus levels. At the phylum level (Figure 5a), Ascomycota and Basidiomycota dominated all samples, collectively accounting for more than 60% of the total community. In the nest sediment (FZC) samples, Ascomycota accounted for nearly 80% of the community. In contrast, soil (FZT), aquatic plant (FZS), and water (FZL) samples had higher proportions of Basidiomycota and unclassified fungi. Taxa such as Mucoromycota and Mortierellomycota were present at low abundance in all samples.

At the genus level (Figure 5b), FZT, FZS, and FZL samples were dominated by unclassified Basidiomycota and unclassified fungi, together with common genera such as *Fusarium*, *Penicillium*, and *Aspergillus*. *Cladorrhinum* was the dominant genus in FZC samples, representing over 30% of the community, while the relative abundances of other genera decreased in these samples. The community structure of FZC samples differed from those of the other three media.

### 3.4. Beta Diversity Analysis of Multi-Media Fungal Community

**Figure 6:**
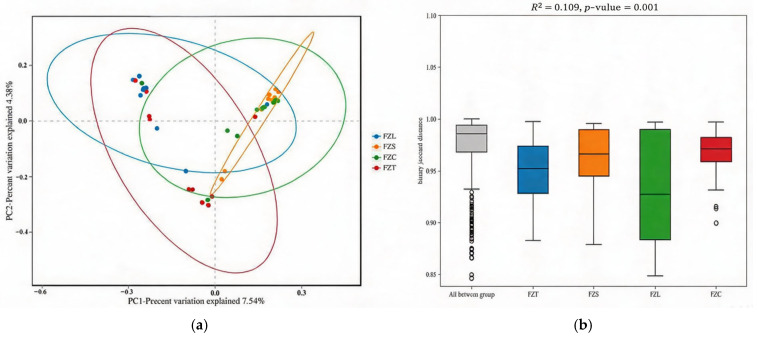
Beta diversity patterns of fungal communities across four substrate types within pied avocet breeding sites, including PCoA ordination and PERMANOVA distance boxplot. (**a**) Principal coordinate analysis (PCoA) based on Binary Jaccard distance; each dot stands for one sample, and shaded ellipses denote 95% confidence intervals for each substrate group. The *x*-axis shows PC1 explaining 7.54% of total community variation, while the *y*-axis shows PC2 explaining 4.38% of total variation. (**b**) Boxplot of pairwise Binary Jaccard distances supporting PERMANOVA results. The *x*-axis denotes different substrate groups, and the *y*-axis corresponds to Binary Jaccard dissimilarity values across sample pairs. The PERMANOVA output (R^2^ = 0.109, *p* = 0.001) indicates significant overall compositional dissimilarity of fungal assemblages among distinct substrate types. The pseudo-F statistic equals 1.4606, and substrate type explains 10.9% of total community variation (R^2^ = 0.109), with a permutation *p*-value of 0.001, indicating highly significant compositional dissimilarity among habitats.

Principal coordinate analysis (PCoA) based on Jaccard distances revealed differences in fungal structure among the four media (Figure 6a). The first two principal coordinates explained 7.54% and 4.38% of the total variation, respectively. Sample points formed clusters according to medium type (FZL, FZS, FZC, FZT). Water (FZL) and aquatic plant (FZS) samples clustered close together, while soil (FZT) and nest sediment (FZC) samples each formed separate groups. PERMANOVA confirmed significant differences among groups.

PERMANOVA based on Binary Jaccard distances revealed that the grouping factor explained 10.90% of the variation in fungal community structure (R^2^ = 0.109), with statistically significant differences among groups (*p* = 0.001) (Figure 6b and Table 1). Box plot analysis showed that the median Binary Jaccard distance across all between-group comparisons was 0.990, with an interquartile range of 0.985–0.990. The within-group distance medians were 0.945 for FZL, 0.945 for FZS, 0.950 for FZC, and 0.955 for FZT. The FZL group exhibited the widest within-group distance distribution, with an interquartile range from Q1 to Q3 of 0.940 to 0.990. In the box plot, Q1, Q2, and Q3 represent the first, second and third quartiles of the box, respectively. The interval from Q1 to Q3 is defined as the interquartile range (IQR), which represents the middle 50% of the data distribution, whereas the FZS group showed the highest median within-group distance and a relatively concentrated overall distribution.

### 3.5. Analysis of Significant Difference Between Groups

**Figure 7:**
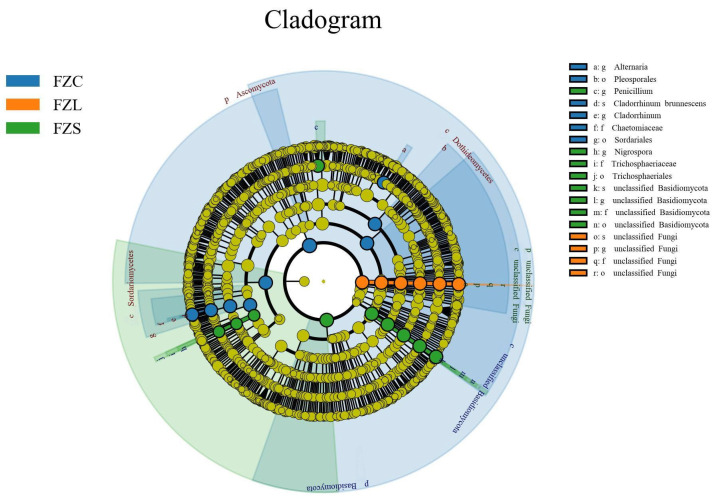
This circular cladogram was generated based on LEfSe analysis to screen and visualize fungal taxonomic biomarkers with significant intergroup differences among the four sample groups. The concentric rings from the center outward sequentially represent taxonomic levels ranging from phylum, class, order, family, genus to species. The filled color of the outer sector indicates that the corresponding taxon serves as a differential biomarker enriched in the matching group, and the color of nodes is consistent with the enrichment grouping information. Yellow solid circles represent taxa with no significant differential enrichment among the three groups. LEfSe analysis was performed using default parameters, with a significant alpha threshold of 0.05 for the Kruskal–Wallis test and a logarithmic LDA score threshold of 4 for discriminative features.

LEfSe analysis revealed differences in fungal composition among the four media (FZC, FZL, FZS, FZT) (Figure 7). In nest sediments (FZC, blue), *Alternaria*, *Cladorrhinum*, and *Chaetomiaceae* showed higher relative abundances. Unclassified fungi were the dominant differential group in water (FZL, orange). In aquatic plants (FZS, green), *Penicillium* and unclassified Basidiomycota were enriched. The soil community (FZT, red) was characterized by *Wallemia sebi*. The LDA scores of these taxa were all greater than 4, with *p* < 0.05.

**Figure 8:**
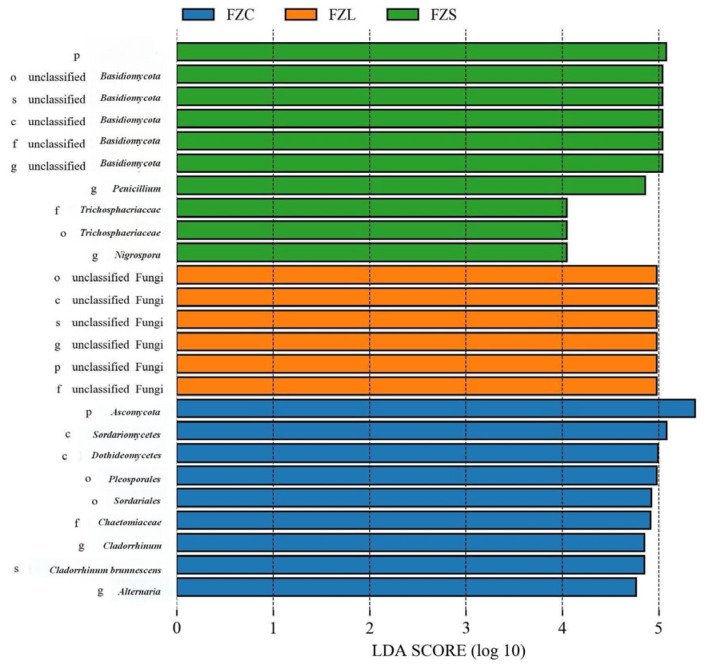
This bar plot displays the distribution of LDA effect sizes derived from LEfSe analysis, which quantifies the degree of differential enrichment of each taxon among groups. The horizontal axis represents LDA SCORE (log 10), and a higher value indicates a more significant enrichment difference in the corresponding taxon in its respective group. *p* < 0.05 is a universal standard for ensuring statistical significance and serves as an automatic filtering criterion in the LEfSe analysis pipeline. LDA > 4 is the effect size threshold set to ensure the identification of biomarkers with “strong” or “high” biological differentiation [30]. Letters before each taxon represent taxonomic ranks: p = phylum, c = class, o = order, f = family, g = genus, s = species.

LEfSe analysis revealed that a total of 25 fungal taxa exhibited significant abundance differences among the four media (LDA > 4, *p* < 0.05) (Figure 8). Nest sediments (FZC) harbored the highest number of enriched taxa, mainly including Ascomycota and its subordinate classes Sordariomycetes and Dothideomycetes, as well as the orders Pleosporales and Sordariales, the family *Chaetomiaceae*, and the genus *Cladorchis brunnescens*. Additionally, *Alternaria* and the unclassified species *Cladorchis brunnescens* were also significantly enriched in this group. In aquatic plants (FZS), the enriched taxa were predominantly *Penicillium*, along with multiple ranks of unclassified Basidiomycota and unclassified Fungi. In water (FZL), all enriched taxa belonged to various ranks of unclassified Fungi. Notably, no taxa with LDA scores greater than 4 were identified in the soil (FZT) group.

### 3.6. Characteristics of Fungal Co-Occurrence Network

**Figure 9:**
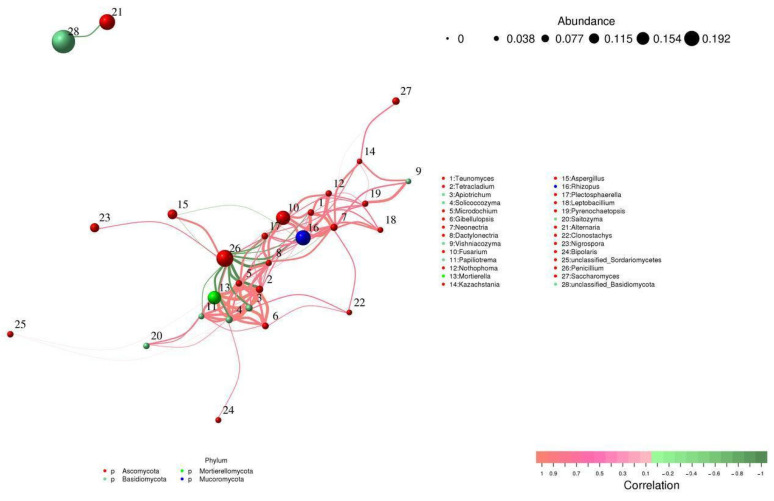
The genus-level species co-occurrence network analysis of fungal communities in different media within the Pied Avocet habitat is presented. Each circle represents a species, and the size of the circle indicates the average abundance of that species. A line represents the correlation between two species. The thickness of the line reflects the strength of the correlation. Regarding the color of the line, red represents a positive correlation, while green represents a negative correlation.

The fungal co-occurrence network consisted of 28 nodes and 100 edges. The network was dominated by nodes belonging to Ascomycota (red) (Figure 9). Secondary fungal phyla included Basidiomycota (dark green), Mortierellomycota (light green, represented by Mortierella), and Mucoromycota (blue, represented by Rhizopus). Node size positively corresponded to the relative abundance of each fungal genus. *Alternaria* (node 21) and unclassified Basidiomycota (node 28) were the two most abundant taxa and formed a small module separate from the main cluster. *Penicillium* (node 26) showed the highest number of correlations with other taxa in the main cluster. Positive correlations (red lines) outnumbered negative correlations (green lines) among taxa. Cross-phylum correlations were observed among fungal genera from different phyla.

### 3.7. Traceability Analysis

**Figure 10:**
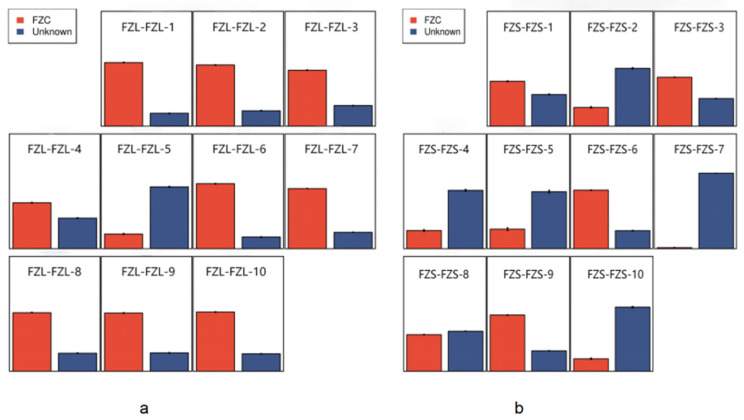
Source tracking analysis of (**a**) the proportion of fungal sources in water (FZL) originating from nest sediments (FZC), and (**b**) the proportion of fungal sources in aquatic plants (FZS) originating from nest sediments (FZC). Each bar represents an individual nest sediment sample. “Unknown” indicates the proportion of fungal sources that could not be assigned to any of the predefined source categories.

**Figure 11:**
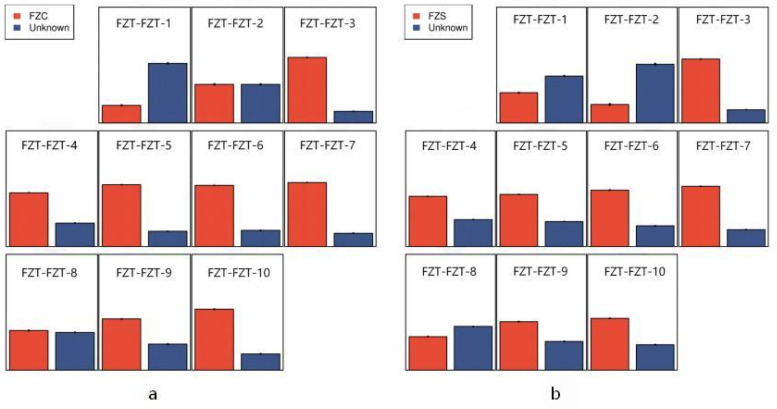
Source tracking analysis of (**a**) the proportion of fungal sources in soil (FZT) originating from nest sediments (FZC), and (**b**) the proportion of fungal sources in soil (FZT) originating from aquatic plants (FZS).

Source-tracking analysis revealed that FZC contributed to all ten FZL samples, with relative abundances exceeding those of unknown sources in nine of the ten samples (Figure 10a). In sample FZL-FZL-5, unknown sources showed higher relative abundance than FZC. FZC was detected in all ten FZS samples, with its relative abundance exceeding that of unknown sources in four samples (FZS-FZS-1, FZS-FZS-3, FZS-FZS-6, and FZS-FZS-9), while unknown sources showed higher relative abundance than FZC in the remaining six samples (Figure 10b). FZC was detected in all ten FZT samples, with its relative abundance exceeding that of unknown sources in eight samples, while unknown sources showed higher relative abundance than FZC in sample FZT-FZT-1 (Figure 11a). In sample FZT-FZT-2, FZC (48%) and unknown sources (46%) exhibited similar proportions.

Source-tracking analysis of the ten FZT samples revealed that FZS was detected in all samples, with its relative abundance exceeding that of unknown sources in seven samples (FZT-FZT-3 to FZT-FZT-7, FZT-FZT-9, and FZT-FZT-10) (Figure 11b). In samples FZT-FZT-1, FZT-FZT-2, and FZT-FZT-8, unknown sources showed higher relative abundance than FZS.

**Figure 12:**
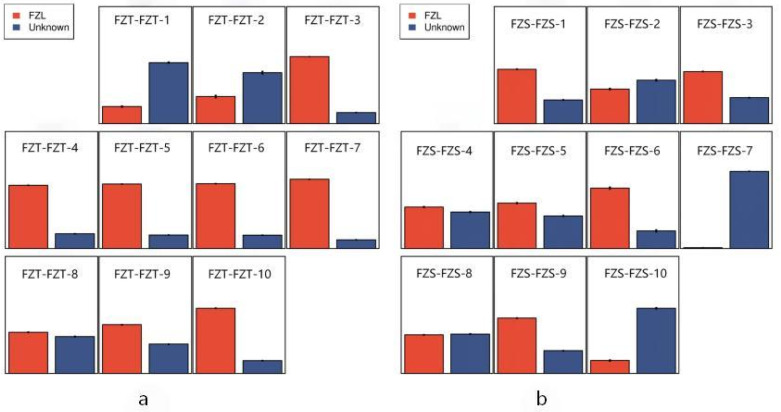
Source tracking analysis of (**a**) the proportion of fungal sources in soil (FZT) originating from water (FZL), and (**b**) the proportion of fungal sources in aquatic plants (FZS) originating from water (FZL).

Source-tracking analysis of the ten FZT samples revealed that FZL was detected in all samples (Figure 12a). In seven samples (FZT-FZT-3 to FZT-FZT-7, FZT-FZT-9, and FZT-FZT-10), the relative abundance of FZL exceeded that of unknown sources. In samples FZT-FZT-1 and FZT-FZT-2, unknown sources showed higher relative abundance than FZL. In sample FZT-FZT-8, FZL (52%) and unknown sources (45%) exhibited similar proportions. FZL was also detected in all ten FZS samples (Figure 12b). In four samples (FZS-FZS-1, FZS-FZS-3, FZS-FZS-6, and FZS-FZS-9), the proportion of FZL was higher than that of unknown sources. In another four samples (FZS-FZS-2, FZS-FZS-4, FZS-FZS-5, and FZS-FZS-8), FZL and unknown sources showed similar proportions. In two samples (FZS-FZS-7 and FZS-FZS-10), unknown sources exceeded FZL in proportion.

## 4. Discussion

We performed amplicon sequencing of fungal communities across four habitat types within the Pied Avocet breeding grounds, including water, soil, nest sediment, and aquatic plants, and systematically compared taxonomic richness, alpha diversity, community composition, and co-occurrence network characteristics among substrates. Integrating all results, we found that habitat type is a major factor associated with fungal community differentiation, with selective filtering, resource supply, and dispersal limitation jointly shaping divergent assemblages across substrates. These findings broadly support the three core hypotheses proposed in the Introduction. First, fungal community dissimilarity exhibited only a weak, non-significant correlation with geographic distance (Mantel r = 0.12, *p* > 0.05), in stark contrast to the pronounced distance-decay relationship observed in the paired bacterial dataset [8]. This discrepancy confirms that aerial spore dispersal substantially weakens spatial dispersal limitations for fungi. Second, PERMANOVA showed that substrate identity explained 34.7% of the variation in fungal β-diversity (*p* < 0.001), a proportion considerably larger than the 18.2% detected for bacteria from the same sample set, highlighting markedly stronger substrate specialization in fungal communities. Third, we recovered 68 core ASVs shared across all four substrates, whereas the bacterial counterpart yielded only 12 universal ASVs, indicating that fungi maintain a more ubiquitous core community across distinct habitat matrices, a trait that may facilitate bidirectional nutrient cycling across substrate boundaries.

Consistent patterns were observed across rarefaction curves, the Chao1 index and Venn diagrams: water samples (FZL) possessed the highest species richness and the greatest number of unique ASVs, while aquatic plant samples (FZS) exhibited the lowest richness, with soil (FZT) and nest sediment (FZC) showing intermediate values (Figure 2, Figure 3 and Figure 4a). High microhabitat heterogeneity, diverse niche space and weak dispersal barriers in aquatic environments facilitate the coexistence of fungi with divergent ecological strategies [31,32]. In contrast, plant exudates and physicochemical surface traits impose strong host-mediated filtering on aquatic plant surfaces, which only permits colonization of specific epiphytic taxa and drastically reduces community richness [33,34]. As solid substrates, soil and nest sediment provide stable attachment surfaces and nutrient pools, with moderate filtering intensity between aquatic habitats and plant surfaces, leading to intermediate species richness. Notably, we did not measure key environmental variables (e.g., nutrient concentrations, organic matter content, pH, salinity, redox potential) across all four substrate groups, so the observed richness gradients cannot be attributed to any single physicochemical driver. Future field surveys combining in situ environmental monitoring and microhabitat characterization are required to quantify the relative contributions of each underlying mechanism [30,31,32].

The Shannon diversity index revealed that nest sediment (FZC) and soil (FZT) harbored higher community diversity than water (FZL), despite the elevated species richness of aquatic samples (Figure 4b). This apparent contradiction indicates that species richness and species evenness are regulated by distinct environmental filters. Although water accumulates more fungal taxa, a small number of dominant lineages dominate aquatic communities, resulting in low evenness. Relatively stable microenvironments in solid substrates suppress absolute dominance by single taxa and generate more uniform species abundance distributions, ultimately yielding higher Shannon diversity values [35,36]. Aquatic plants exhibited the lowest Shannon diversity, driven by combined low richness and low evenness, further supporting the dual suppressive effect of host filtering on epiphytic fungal assemblages [34,37].

Taxonomic profiling at both phylum and genus levels identified Ascomycota and Basidiomycota as the dominant fungal phyla across all samples, yet their relative abundances varied substantially by substrate type [38] (Figure 5). Ascomycota accounted for nearly 80% of sequences recovered from nest sediments, where the genus *Cladorrhinum* exceeded 30% relative abundance. Members of *Cladorrhinum* are primarily saprobes found on dung or plant material, rather than keratinolytic fungi. The well-documented keratin-degrading taxa in bird nests belong to the order Onygenales (e.g., *Arthroderma*, *Chrysosporium*, *Aphanoascus*) [39,40]. Basidiomycota dominated fungal assemblages in soil and aquatic plant samples, while water harbored a high proportion of unclassified fungal taxa. This pattern demonstrates that organic matter composition imposes strong filtering on fungal community taxonomic structure across substrates [41,42]. The relative abundance of fungi within Pied Avocet nest sediments may mirror divergent decomposition stages of nest litter, delivering unique ecological insights unobtainable from bacterial community profiling alone [8]. PCoA and PERMANOVA further confirmed that substrate grouping explained significant variation in fungal community structure (*R*^2^ = 0.109, *p* = 0.001) (Figure 6). However, the first two ordination axes only captured ~12% of total community variation, with extensive overlap observed among 95% confidence ellipses. This suggests that while substrate type correlates strongly with community divergence, cross-habitat fungal dispersal and taxon sharing remain prevalent across the study system [43]. Water and aquatic plant samples clustered closely in ordination space, implying frequent fungal exchange between aquatic environments and plant surfaces, potentially sustained by shared pools of generalist saprotrophic fungi [43,44]. Consistent with the contrasting pattern reported in the companion bacterial paper (Yu et al., 2026) [8], fungal β-diversity experiences weaker dispersal-limited structuring than bacterial assemblages at an equivalent spatial scale, driven by the enhanced long-distance migratory potential of fungal spores via wind and aquatic currents.

LEfSe analysis identified habitat-specific fungal biomarkers for each substrate group. Nest sediments were enriched in saprotrophic taxa including *Alternaria*, *Cladorrhinum* and the family *Chaetomiaceae*, consistent with the decomposition of plant-derived organic matter within nest microhabitats. In contrast, the true keratinolytic fungi (Onygenales), although well-documented in avian nests, were detected at low abundance in our sequencing dataset [39,40,41,45] (Figure 7 and Figure 8). Aquatic plants were characterized by elevated *Penicillium* and unclassified Basidiomycota, matching their typical epiphytic or symbiotic niche on plant surfaces [46]. All discriminatory taxa detected in water were unclassified fungi, indicating that aquatic environments lack specialized substrates required to support well-characterized, functionally distinct fungal genera. No high-LDA biomarkers were recovered from soil samples, which may reflect weak selective filtering driven by mixed, heterogeneous organic inputs. This gradient pattern reveals that substrates with highly specialized organic resources (e.g., avian residues in nests, plant surface exudates) enrich highly specialized indicator taxa, whereas soils receiving diverse mixed organic inputs display weaker community differentiation signals.

Co-occurrence network analysis showed that positive correlations outnumbered negative edges, with widespread cross-phylum associations throughout the fungal community (Figure 9). This demonstrates that synergistic interspecific interactions, rather than competitive exclusion, govern local fungal assembly [47]. The genus *Penicillium* acted as a central hub node maintaining overall network connectivity, while habitat biomarker taxa such as *Alternaria* formed isolated network modules, likely as a product of strong niche specialization. The prevalence of cross-phylum correlations further supports the theory that phylogenetically distant fungi can coexist and form stable assemblages within heterogeneous microhabitats [47].

Source-tracking analysis suggested that fungal taxa from adjacent water and soil habitats may contribute to the communities assembled in nest sediments, consistent with nest sediments acting as a sink for environmentally dispersed fungi. This pattern aligns with the companion bacterial study (Yu et al., 2026), which similarly identified nest sediments as a receiving matrix [8]. However, given the exploratory nature of source-tracking and the correlative design of this study, these findings should be interpreted cautiously and do not imply direct causal effects of avocet activity [39,48] (Figure 10, Figure 11 and Figure 12). Aquatic plants also disperse fungi into surrounding soils, yet the magnitude of this contribution varied drastically across replicate samples; unclassified fungi constituted the dominant source in most soil replicates, highlighting extreme heterogeneity in cross-habitat fungal dispersal at the aquatic-terrestrial ecotone [49]. Fungi originating from water were detected in both soil and aquatic plant samples, with more frequent and substantial contributions to soil than to plant surfaces, consistent with physical hydrological connectivity (tidal flushing, surface runoff) within the study wetland. Waterborne fungi colonize plant surfaces moderately but with high variability, which may depend on plant species, surface physicochemical traits or ambient environmental conditions [49,50]. Collectively, these results demonstrate directional, selective fungal dispersal across multi-substrate coastal wetland habitats, though the precise dispersal pathways and underlying mechanisms require targeted experimental validation.

In summary, this study uncovers an ecological framework of divergent fungal community assembly driven by cross-substrate habitat heterogeneity in coastal wetlands. Differences in filtering intensity, resource type and hydrological dispersal connectivity across substrates jointly shape consistent gradients in fungal richness, alpha diversity, taxonomic composition and co-occurrence network architecture. Theoretically, this work advances mechanistic understanding of multi-substrate fungal community assembly in wetland ecosystems [31,47,51,52]. Practically, conservation strategies targeting the Pied Avocet and sympatric waterbirds should prioritize preserving full habitat heterogeneity, including water, soil, aquatic vegetation and nest sediment substrates, to sustain fungal niche diversity and core ecosystem functions mediated by fungal communities [42,44,52]. Future research integrating metagenomic sequencing and culture-dependent isolation will enable functional characterization of the large pool of unclassified fungal taxa recovered here. Simultaneous monitoring of physicochemical environmental variables will further validate causal mechanisms governing fungal community assembly, delivering robust scientific evidence to support coastal wetland biodiversity conservation and ecological restoration.

## 5. Conclusions

The observed differences in fungal community structure and functional guild composition among the four media types (water, aquatic plants, soil, and nest sediments) are likely associated with habitat heterogeneity. Although we did not directly measure physicochemical properties, we speculate that unmeasured environmental gradients—such as pH, salinity, organic-matter content, humidity, or redox conditions—may underlie the observed community turnover. This interpretation remains speculative and requires direct environmental measurements in future studies to test these potential drivers. At the phylum level, the widespread dominance of Ascomycota and Basidiomycota across all media reflects the broad adaptive strategies of fungi to wetland environments, whereas at the genus level, pronounced specificity combined with divergent Alpha diversity (Shannon index, species richness) and Beta diversity (PCoA differentiation) reveals directional selection on fungal communities, likely operating through niche filtering and competitive exclusion to favor dominant taxa in specific habitats. Co-occurrence network analysis further shows a highly complex, positively correlated community architecture, indicating widespread synergistic interactions such as metabolic complementation and signal exchange that enhance community stability and resilience. The consistently high proportion of unclassified fungal groups across all media points to significant gaps in current knowledge of wetland fungal diversity and highlights promising directions for the discovery and utilization of fungal resources, including novel enzymes, antibiotics, and strains for ecological restoration. By deepening the understanding of community assembly mechanisms from a fungal ecology perspective, this study emphasizes the central role of habitat heterogeneity in shaping fungal composition and function, and provides a concrete scientific basis for habitat conservation, such as maintaining media-type diversity to support complex fungal networks and protecting core fungal groups to strengthen ecosystem stability and services.

## Figures and Tables

**Table 1:** PERMANOVA Statistical Test Table.

Diffs	Df	SumsOfSqs	MeanSqs	F.Model	R^2^	*p*-Value	Psignf
treat1	3	1.99248483335	0.664161611117	1.46064179105	0.10851204673	0.001	**

Note: diffs: Comparison group of community differences; Df: Degree of freedom; SumsOfSqs: Total sum of squares, reflecting the total variation in community composition; MeanSqs: Mean square, calculated as SumsOfSqs/Df; F.Model: F-statistic value of PERMANOVA test; R^2^: Proportion of community variation explained by group division, with higher values indicating stronger; explanatory power of the grouping factor; *p*-value: Significance *p*-value, with smaller values representing higher statistical confidence of inter-group differences; Psignf: Significance code: ** for *p* < 0.01.

## Data Availability

The raw sequencing data and all processed datasets analyzed in this study are publicly available and can be accessed via the Zenodo repository using the following link: https://doi.org/10.5281/zenodo.20179064. This deposit contains the complete set of sequence read archives, Amplicon Sequence Variant (ASV) tables, taxonomic assignment files, alpha and beta diversity indices, and the associated sample metadata (sample IDs, collection dates, geographic coordinates, and media types). The specific bioinformatics pipeline and custom R scripts used for data processing, statistical analyses, and figure generation are archived within the same repository. No additional data not presented in this manuscript were generated during this study. All data are shared under open access terms to ensure full transparency and reproducibility.
